# Advanced Running Performance by Genetic Predisposition in Male Dummerstorf Marathon Mice (DUhTP) Reveals Higher Sterol Regulatory Element-Binding Protein (SREBP) Related mRNA Expression in the Liver and Higher Serum Levels of Progesterone

**DOI:** 10.1371/journal.pone.0146748

**Published:** 2016-01-22

**Authors:** Daniela Ohde, Mark Moeller, Julia Brenmoehl, Christina Walz, Siriluck Ponsuksili, Manfred Schwerin, Georg Fuellen, Andreas Hoeflich

**Affiliations:** 1 Institute of Genome Biology, Leibniz Institute for Farm Animal Biology (FBN), Dummerstorf, Germany; 2 Institute for Biostatistics and Informatics in Medicine and Ageing Research, Rostock University Medical Center, Rostock, Germany; University of Basque Country, SPAIN

## Abstract

Long-term-selected DUhTP mice represent a non-inbred model for inborn physical high-performance without previous training. Abundance of hepatic mRNA in 70-day male DUhTP and control mice was analyzed using the Affymetrix mouse array 430A 2.0. Differential expression analysis with PLIER corrected data was performed using AltAnalyze. Searching for over-representation in biochemical pathways revealed cholesterol metabolism being most prominently affected in DUhTP compared to unselected control mice. Furthermore, pathway analysis by AltAnalyze plus PathVisio indicated significant induction of glycolysis, fatty acid synthesis and cholesterol biosynthesis in the liver of DUhTP mice versus unselected control mice. In contrast, gluconeogenesis was partially inactivated as judged from the analysis of hepatic mRNA transcript abundance in DUhTP mice. Analysis of mRNA transcripts related to steroid hormone metabolism inferred elevated synthesis of progesterone and reduced levels of sex steroids. Abundance of steroid delta isomerase-5 mRNA (Hsd3b5, FC 4.97) was increased and steroid 17-alpha-monooxygenase mRNA (Cyp17a1, FC -11.6) was massively diminished in the liver of DUhTP mice. Assessment of steroid profiles by LC-MS revealed increased levels of progesterone and decreased levels of sex steroids in serum from DUhTP mice versus controls. Analysis of hepatic mRNA transcript abundance indicates that sterol regulatory element-binding protein-1 (SREBP-1) may play a major role in metabolic pathway activation in the marathon mouse model DUhTP. Thus, results from bioinformatics modeling of hepatic mRNA transcript abundance correlated with direct steroid analysis by mass spectrometry and further indicated functions of SREBP-1 and steroid hormones for endurance performance in DUhTP mice.

## Introduction

Higher endurance performance can be acquired by physical training [1] and further improved by specific diet programs [2]. In addition, higher endurance capacities can also be conferred by genetic predisposition [3]. It is well known that physical activity results in numerous adaptations in muscle [4, 5], heart [6], fat [7], respiratory system [8], and many other body tissues, including the brain [9, 10]. Only a limited number of polymorphisms e.g., myostatin [11] has been associated with superior endurance performance and discussed as potential selection criteria for young human athletes. Different rodent models have been established for the study of genetic disposition on endurance exercise [12, 13]. The mouse model [12] was selected based on high voluntary running wheel activity in the presence of running wheels during selection. As a consequence, these mice can be used to study interactions of physical activity, home cage activity, and genetic predisposition [14, 15]. The rat model was specifically developed by applying a complex training program before the estimation of maximal endurance running capacities [13]. In *Thoroughbred* horses, as a large animal model, a genome scan revealed a number of genes relevant for fatty acid metabolism, insulin sensitivity, and muscle strength associated with endurance performance [16]. Besides effects on muscle structure and function, metabolic adaptations in muscle and adipose tissues may be related to superior running performance in horses [16], and similar observations were made for mice [17] and humans [18]. During physical exercise, hepatic lipid metabolism is affected by training intensity and physical condition in humans and rodent models [19]. Moreover, physical exercise has been discussed as a means to modulate lipid metabolism as an intervention strategy against fatty liver disease and obesity [19].

We have previously reported a mouse model supporting an intricate link between running capacity and hepatic lipid metabolism that could be used in order to study control of metabolic fluxes in the liver [20]. This mouse model (DUhTP) had been previously established by long-term selection for high treadmill performance [21]. DUhTP mice are characterized by 4-fold increased running performance with no previous physical training, as the selection experiment had been performed in the absence of running wheels. Sedentary DUhTP mice have high levels of hexose and lipids in their livers as exhibited by a metabolome analysis [20]. In the present study, we examined hepatic mRNA transcript abundance in our mouse model to gain additional insights into the metabolic pathway control associated with advanced physical performance of DUhTP mice. In particular we asked whether the results from the analysis on the mRNA level 1) support findings from proteomic or metabolic studies in the same animal model and 2) can be used in order to investigate the control of energy metabolism in the liver of DUhTP mice.

## Materials and Methods

### Animals

The Dummerstorf mouse line DUhTP used in the present study has been generated by long term phenotype selection for high treadmill performance over 95 generations [20, 21]. All procedures were done in accordance to national and international guidelines and approved by our own institutional board (Animal Protection Board from the Leibniz-Institute for Farm Animal Biology) and by the national Animal Protection Board Mecklenburg-Vorpommern (file number: LALLF M-V/TSD/7221.3–1.2-037/06). For the study we used 10 male mice each from DUhTP and control (DUC) at an age of 10 weeks. Immediately after decapitation defined lobes from the liver were removed, snap frozen, ground in liquid nitrogen to a homogenous powder and stored at -80°C for subsequent analysis.

### RNA preparation

Total RNA was isolated from snap frozen, powdered liver using 1 ml TRI Reagent (Sigma-Aldrich, Taufkirchen, Germany) and syringes and needles (21G x 1½”) according to the manufacturer’s protocol. After DNase I treatment the RNA was further purified using the RNeasy Kit (Quiagen, Hilden, Germany). RNA quantity was determined using the NanoDrop ND-1000 spectrophotometer (Peqlab, Erlangen, Germany) and the integrity was checked by gel electrophoresis of 1μg RNA in 1% agarose.

### Gene expression profiling

Gene expression in the mouse liver was assessed using the mouse genome 430A 2.0 array (Affymetrix, Santa Clara, CA). For the preparation of target products 5 μg of total RNA were included. Hybridization and scanning on a GeneChip scanner 3000 (Affymetrix) were performed according to the manufacturer’s protocols. Data were analyzed using Affymetrix GCOS 1.1.1 software and global scaling to a target signal of 500. Data were imported into Array Assist software (Stratagene) and processed with MAS (Microarray Suite) 5.0 to generate cell intensity files (Affymetrix 2001). Quantitative expression levels of the present transcripts were estimated using PLIER (Probe Logarithmic Intensity Error, Affymetrix 2005) for normalization. The results have been submitted to Gene Expression Omnibus (GEO submission number GSE73539).

### Microarray preprocessing and identification of differentially abundant mRNA

We used PLIER corrected expression data (GeneChip Mouse Genome 430A 2.0 Array) of three pools per strain (DUhTP, DUC). The GeneChip Mouse Genome 430A 2.0 Array includes 22,600 probe sets associated to roughly 14,000 genes. The probe sets belonging to the same gene were treated independently. Differential analysis was performed with AltAnalyze v.3.1.2 [22]. The raw p-value was calculated by a moderated unpaired t-test for each pairwise comparison. The original raw p-values were corrected for multiple testing by applying the Benjamini Hochberg algorithm (i.e. false discovery rate/FDR; [23]). Unless stated otherwise, probe sets were classified as significantly differentially expressed if they featured an adjusted p-value below 0.05 and a fold change (FC) larger than 2. Visualization was achieved by PathVisio v.2.0.8 [24]. The online tool Panther v.10.0 was used for functional classification of the differentially expressed genes [25] based on Gene Ontology terms [26].

Functional analysis was also performed using AltAnalyze v.3.1.2. Specifically, we searched for over-representation in biochemical pathways (KEGG [27], PathwayCommons [28], WikiPathways [29]), and Gene Ontology terms [30]. PathVisio was used to map the gene expression data to reaction pathways of WikiPathways.

### Quantitative real-time RT-PCR (qRT-PCR)

Differential mRNA transcript abundance of selected genes was determined by Real time PCR using BioRad iCyler instrumentation (BioRad, Munich, Germany) in technical duplicate reactions. First-strand cDNA was synthesized from total RNA (100 ng/reaction) using RT (SuperScript II, Invitrogen) and oligo(dT)_20_ primers. Gene specific primers were designed using Primer 3 (S1 Table) to amplify PCR products. The reaction mixture consisted of cDNA, 5 μM forward and reverse primers and iCycler reaction mix using manufacturer’s instructions. The template was amplified during 45 cycles at 95°C for 15 seconds (denaturation), 60°C for 10 seconds (annealing) and 72°C for 15 seconds (extension) preceded by initial denaturation at 95°C for 10 min as a universal thermal cycling parameter. Based on the analysis of melting curves of the PCR products a high temperature, fluorescence acquisition point was estimated and included to the amplification cycle program. For all transcripts tested a standard curve was generated by amplifying serial dilutions of specific PCR products. Normalization of variation in RT-PCR efficiency and initial RNA input was performed by using Hprt1 mRNA as internal standard (S2 Table). Data were analyzed using paired t-test and differences were considered significant at p < 0.05.

### Steroid analysis by liquid chromatography coupled to mass spectrometry (LC-MS)

Progesterone, corticosterone, estradiol, and testosterone were assessed in serum from DUhTP mice and controls, as described previously [31]. Serum samples (100 μl) were diluted in 600 μl of methanol/acetonitrile/acetone (1:1:1) and the precipitate was removed by centrifugation for 15 minutes at 4°C. Dried supernatants were stored at -20°C [32–34]. For LC-MS/MS analysis, samples were dissolved in NaHCO_3_ buffer solution (pH 10.5) containing dansyl chloride (1 mg/ml). Derivatization was abrogated on ice and dexamethasone as an internal standard was added at a final concentration of 100 ng/ml [35–37]. LC-MS analysis was performed using an Accela HPLC system (Thermo Fisher Scientific, Dreieich, Germany) coupled to the LTQ Orbitrap high-resolution hybrid mass spectrometer (Thermo Fisher Scientific). For each analyte, a set of seven standard analytes (ranging between 0 and 1,000 ng/ml) was added to an analyte-free matrix in order to establish calibration samples using dexamethasone as an internal calibrator standard to correct for instrumental variations. Curve fitting was performed by nonlinear regression (third order polynomial function).

## Results

### Statistical analysis

From a total of 22,690 probe sets present on the Affymetrix 430A Gene Chip 337 probe sets indicated differential mRNA transcript abundance (p < 0.05) with a fold change (FC) > 2 in long-term selected marathon (DUhTP) mice compared to controls (DUC). Gene symbols could be allocated to 332 of the 337 probe sets. Since several probe sets can refer to the same gene, the total amount of differentially abundant mRNA transcripts is 257 (118 mRNAs were upregulated and 140 mRNAs were down-regulated). The H2-D1 gene is represented by two probe sets, indicating opposite expression levels. GO term analysis with PANTHER [25] revealed 374 functional classification hits enriched in 14 biological processes. The most frequent functional hits refer to metabolic process (GO:0008152) (Table 1).

**Table 1:** Annotation of extracted genes (FC>2, p<0.05) to biological functions (PANTHER). The hash (#) is short for “number of”.

GO biological process		Percent of gene hit against total	
	# genes	genes	function hits
metabolic process (GO:0008152)	125	54.80%	33.40%
cellular process (GO:0009987)	69	30.30%	18.40%
biological regulation (GO:0065007)	39	17.10%	10.40%
localization (GO:0051179)	36	15.80%	9.60%
response to stimulus (GO:0050896)	24	10.50%	6.40%
developmental process (GO:0032502)	20	8.80%	5.30%
immune system process (GO:0002376)	19	8.30%	5.10%
cellular component organization or biogenesis (GO:0071840)	13	5.70%	3.50%
multicellular organismal process (GO:0032501)	10	4.40%	2.70%
apoptotic process (GO:0006915)	5	2.20%	1.30%
reproduction (GO:0000003)	5	2.20%	1.30%
biological adhesion (GO:0022610)	5	2.20%	1.30%
locomotion (GO:0040011)	3	1.30%	0.80%
cell killing (GO:0001906)	1	0.40%	0.30%

### Array data verification by quantitative real-time PCR

In order to validate the microarray data, 15 mRNA transcripts identified by microarray analysis as being differentially abundant were further analyzed by real-time RT-PCR (supporting data). With the exception of heat shock protein 1A (Hspa1a), all mRNA transcripts examined showed the concordant up- or down-regulation in both mouse lines (Table 2).

**Table 2:** Comparison of microarray data and results of qRT-PCR-analysis of liver mRNA. The table represents fold changes (FC) of mRNA abundance in DUhTP versus DUC mice either by microarray or qRT-PCR (*p<0.05, t-test; PP: polypeptide; PK: protein kinase).

Probe Set ID	Gene Title	Gene Symbol	FC (Microarray)	FC (qRT-PCR)
1448319_at	aldo-keto reductase family 1 member B3	Akr1b3	2.41*	2.18*
1455869_at	calcium/calmodulin-dependent PK II	Camk2b	175.52*	1.50*
1435275_at	cytochrome c oxidase subunit Vib, PP 2	Cox6b2	12.19*	18.59*
1419590_at	cytochrome P450, family 2, subfamily b, PP 9	Cyp2b9	14.21*	177.54*
1431803_at	cytochrome P450, family 2, subfamily d, PP 13	Cyp2d13	-1.88	-1.52
1422533_at	cytochrome P450, family 51	Cyp51	2.31*	2.73*
1417991_at	Deiodinase, Iodothyronine, type I	Dio1	1.75	3.79*
1427474_s_at	glutathione S-transferase, mu 3	Gstm3	2.58*	6.78*
1423566_a_at	heat shock protein 110	Hsp110	-1.65	-1.51
1452388_at	heat shock protein 1A	Hspa1a	2.13	-6.81
1422891_at	histocompatibility 2, class IIantigen E alpha	H2-Ea	11.62*	8.28*
1450170_x_at	histocompatibility 2, K1, K region	H2-K1	8.05	1.02
1451239_a_at	solute carrier family 26, member 1	Slc26a1	2.25*	2.14*
1415993_at	squalene epoxidase	Sqle	3.17*	2.75*
1452426_x_at	zinc finger protein 236	Zfp236	5.15	1.19

### Pathway analysis

Over-representation analysis of mRNA abundance identified by 337 different probe sets identified several pathways indicating cholesterol biosynthesis as the most important target (Table 3). Since DUhTP displays an obese phenotype, we furthermore focused on the regulation of glycolysis, gluconeogenesis, fatty acid biosynthesis, and steroid metabolism in DUhTP mice versus controls.

**Table 3:** Top results of enrichment analysis employing PathVisio (s: number of elements (i.e. genes and metabolites) that pass the criterion [FC>2, adj p<0.05]. n: number of elements measured in the experiment. total: total number of elements in the WikiPathway under consideration. sorted by Z-score).

Pathway	Changed	Measured	Set-Number	Changed	Present	Z Score	p-value
	(s)	(n)	(total)	(%[s/n])	(%[n/total])		(permuted)
Cholesterol Biosynthesis (WP103)	8	15	30	53.33%	50.00%	13.1	0
Glucocorticoid and Mineralocorticoid Metabolism (WP495)	5	13	27	38.46%	48.15%	8.64	0
Steroid Biosynthesis (WP55)	5	13	25	38.46%	52.00%	8.64	0
Oxidation by Cytochrome P450 (WP1274)	5	30	49	16.67%	61.22%	5.22	0
Phase I Biontransformations, non P450 (WP1255)	2	7	10	28.57%	70.00%	4.6	0.002
Complement Activation, Classical Pathway (WP200)	3	15	19	20.00%	78.95%	4.54	0.005
Adipogenesis Genes (WP447)	10	128	133	7.81%	96.24%	4.17	0.001
Heme Biosynthesis (WP18)	2	9	22	22.22%	40.91%	3.96	0.004
Leptin and Adiponectin (WP683)	2	9	14	22.22%	64.29%	3.96	0.007
Statin Pathway (WP1)	3	19	29	15.79%	65.52%	3.89	0.003

### Glycolysis and gluconeogenesis

**Fig 1:**
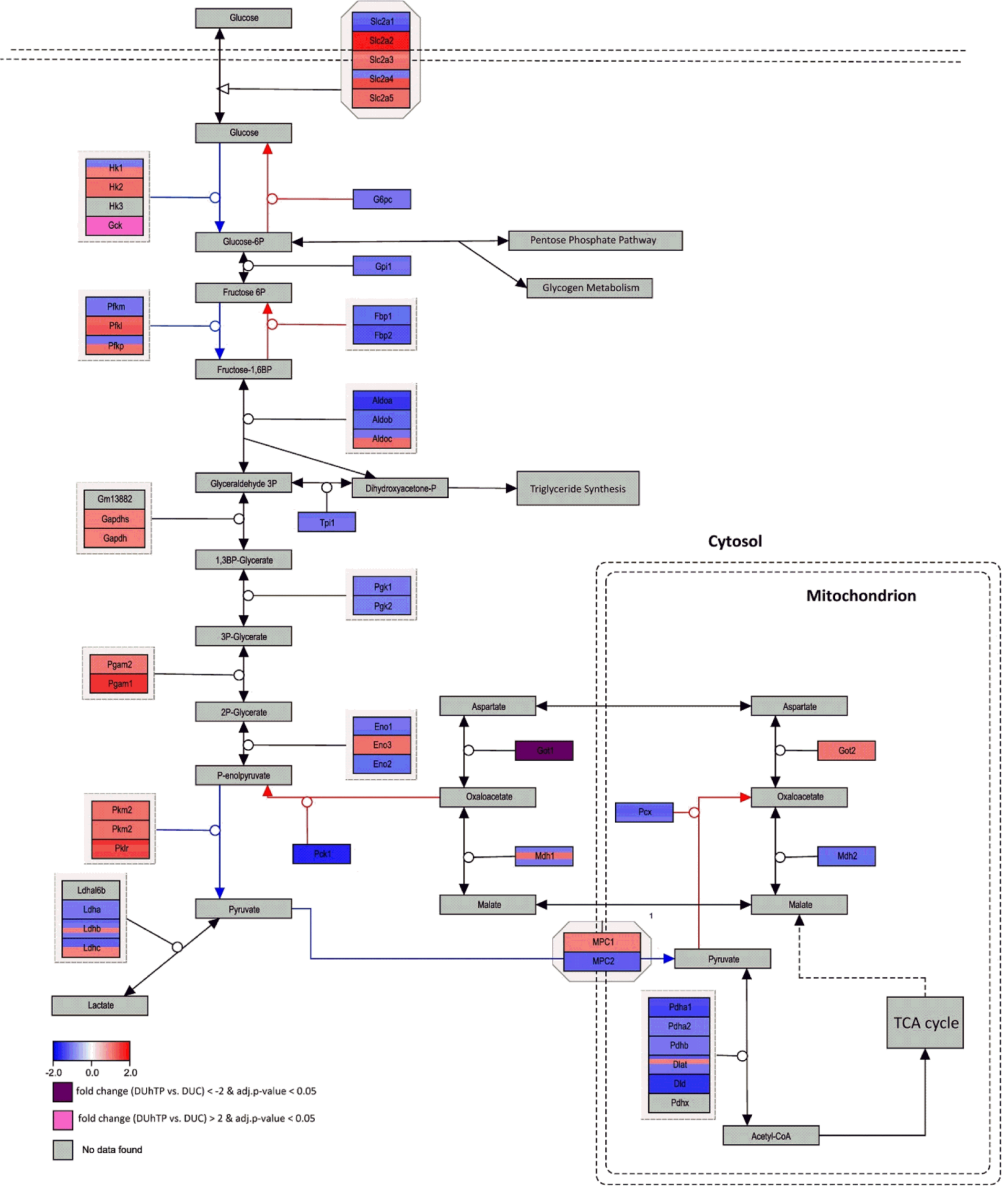
Pathway analysis of microarray results related to Glycolysis and Gluconeogenesis (Mus musculus, WikiPathway WP157) in the liver of DUhTP versus DUC mice. Enzymatic reactions are marked by arrows. Blue arrows indicate specific glycolytic, red arrows specific gluconeogenic and black arrows enzymatic reactions for both glycolysis and gluconeogenesis. Enzyme encoding genes in boxes are colored red or blue, respectively, to indicate up- or down-regulation in DUhTP compared to control DUC. Different colors in a box for the same gene indicate various probe sets with differing expression. Two mRNA transcripts (GCK, GOT1) with fold change >2 are colored in pale and dark magenta for up- or down-regulation, respectively.

We investigated expression of RNA transcripts encoding proteins both for the biosynthesis and breakdown of glucose (Fig 1). From a total of 51 mRNA transcripts involved in the glycolytic/gluconeogenic pathway, expression levels from 47 mRNA transcripts were available. In DUhTP mice versus DUC controls, significant (p<0.05) down-regulation was found for solute carrier family 2, member 1 (glucose transporter, Slc2a1; FC -1.37), phosphoenolpyruvate-carboxykinase (Pck1; FC -1.73), pyruvate-dehydrogenase E1α (Pdha1; FC -1.44) and glutamate oxaloacetate transaminase (Got1; FC -3.83). In addition, four mRNA transcripts were present at higher levels in DUhTP mice versus controls (p<0.05): solute carrier family 2, member 2 (glucose transporter, Slc2a2; FC 1.78), pyruvate kinase (Pklr; 1.58-fold), phosphoglycerate mutase 1 (Pgam1; FC 1.58) and glucokinase (also hexokinase 4, Gck; FC 2.86). Expression levels for other mRNA transcripts from the WikiPathway “glycolysis and gluconeogenesis” (Fig 1) were different in both mouse lines with borderline significance (Slc2a4 p = 0.06; FC 1.42, Aldoa p = 0.058; FC -1.55, Dld p = 0.056; FC -1.58). Since expression of gluconeogenic Pck1 was decreased, whereas glycolytic Gck and Pklr were increased, it seems that the glycolytic pathway is activated, whereas gluconeogenesis is partially inactivated in DUhTP mice versus controls.

### Fatty acid biosynthesis

**Fig 2:**
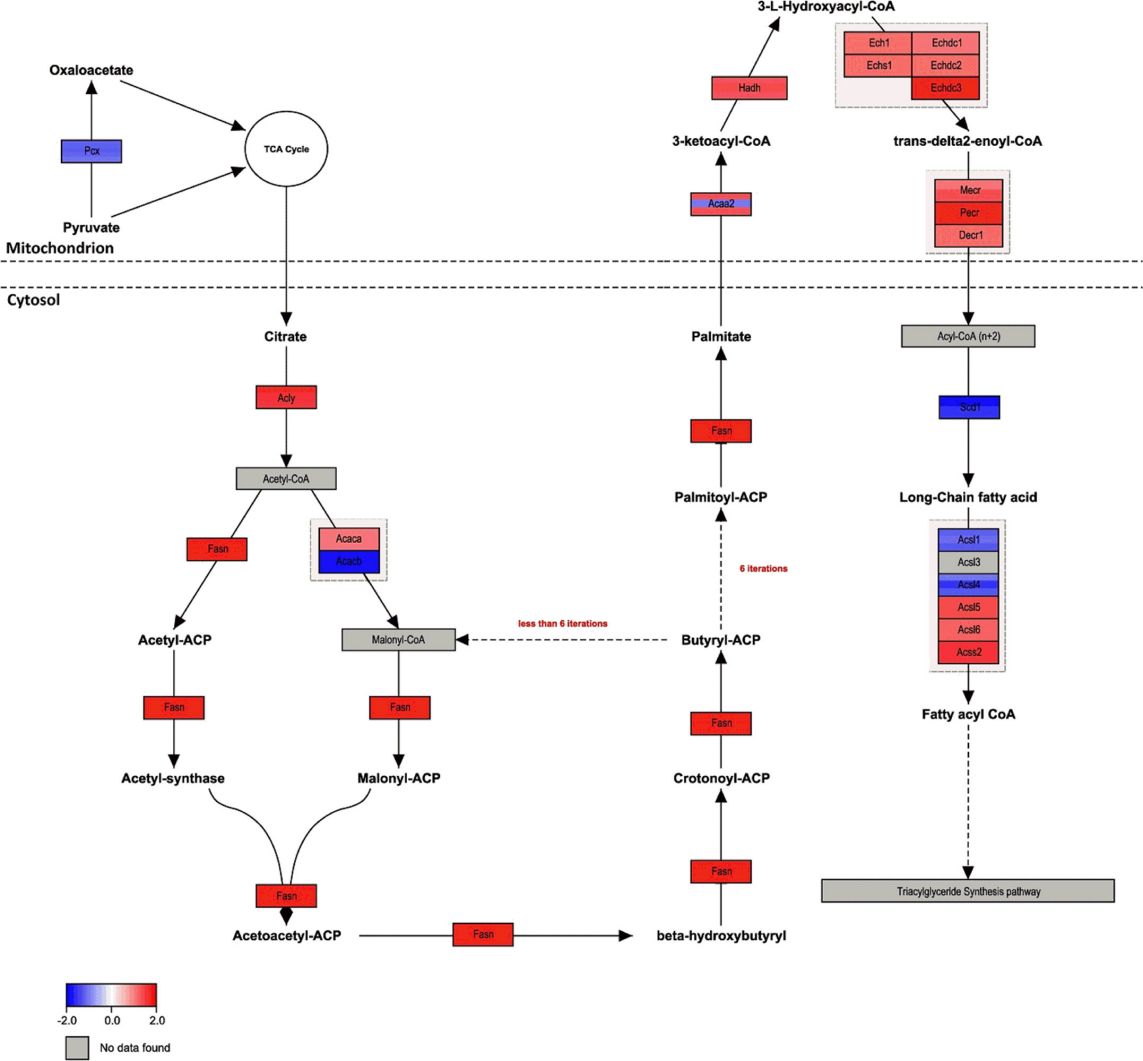
Pathway analysis of microarray results related to Fatty Acid Biosynthesis (Mus musculus, WikiPathway WP336) in the liver of DUhTP versus DUC mice. Enzymatic reactions are marked by arrows. Enzyme encoding genes in boxes are colored red or blue, respectively, to indicate up- or down-regulation in DUhTP compared to control DUC. Different colors in a box for the same gene indicate various probe sets with differing expression. No up- or down-regulation with fold change >2 is found for this pathway.

The gene set of WikiPathway *Fatty Acid Biosynthesis* consists of 23 genes whereof 22 were investigated in this study (Fig 2). From a total of nine differentially expressed mRNA transcripts, the abundance was increased for seven but decreased for two mRNA transcripts. Significantly increased abundance was identified for ATP citrate lyase (Acly; FC 1.69), fatty acid synthase (Fasn, FC 1.75), hydroxyacyl-CoA-dehydrogenase (Hadh, FC 1.36), enoyl-CoA-hydratase domain containing 3 (Echdc3, FC 1.71), peroxisomal trans-2-enoyl-CoA-reductase (Pecr, FC 1.7), as well as for acyl-CoA-synthases long-chain family member 5 (Acsl5, FC 1.38) and short-chain family member 2 (Acss2, FC 1.57) mRNA. Acetyl-CoA-carboxylase beta (Acacb, FC -1.81) and stearoyl-CoA-desaturase 1 (Scd1, FC -1.54) were present at significantly decreased mRNA transcript abundance in DUhTP compared to the unselected control (Fig 2).

### Cholesterol biosynthesis

**Fig 3:**
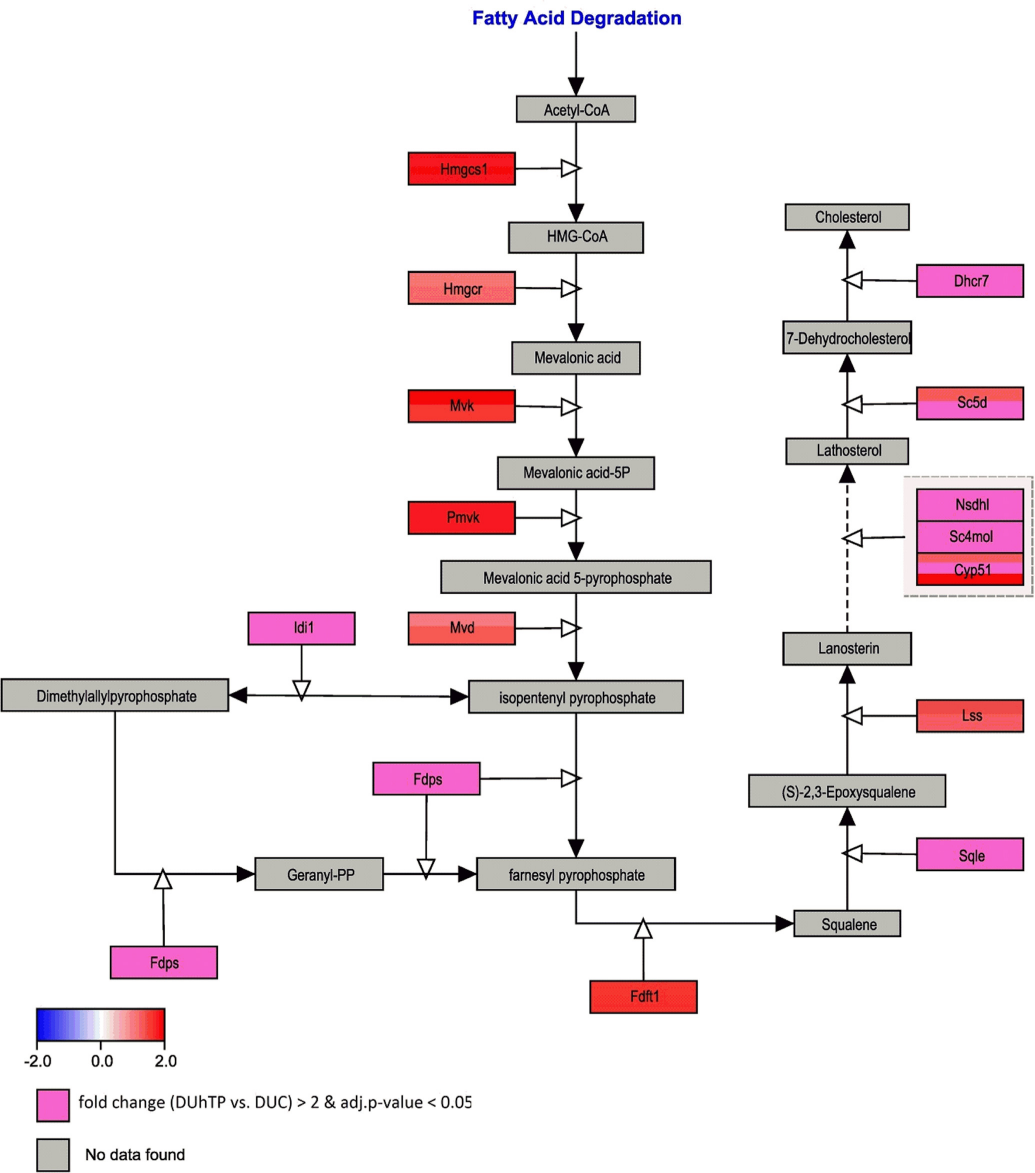
Pathway analysis of microarray results related to Cholesterol Biosynthesis (Mus musculus, WikiPathway WP103) in the liver of DUhTP versus DUC mice. Enzymatic reactions are marked by arrows. Enzyme encoding genes in boxes are colored red or blue, respectively, to indicate up- or down-regulation in DUhTP compared to control DUC. Different colors in a box for the same gene indicate various probe sets with differing expression. Genes with up-regulated fold change >2 are colored in pale magenta.

The WikiPathway gene set entitled *Cholesterol Biosynthesis* covers a total of 15 different genes. With the exception of HMG-CoA-reductase (Hmgcr) and mevalonate-5P-decarboxylase (Mvd), all gene sets revealed significantly increased (p<0.05) abundance of mRNA transcripts in DUhTP mice if compared to unselected controls (Fig 3). Beyond all other gene sets from the WikiPathway *Cholesterol Biosynthesis*, isopentenyl pyrophosphate isomerase mRNA had the strongest increase in the liver from DUhTP mice versus controls (Idi1, FC 3.98). Based on mRNA transcript abundance it is concluded that cholesterol biosynthesis is strongly upregulated in DUhTP mice.

### Steroid biosynthesis

**Fig 4:**
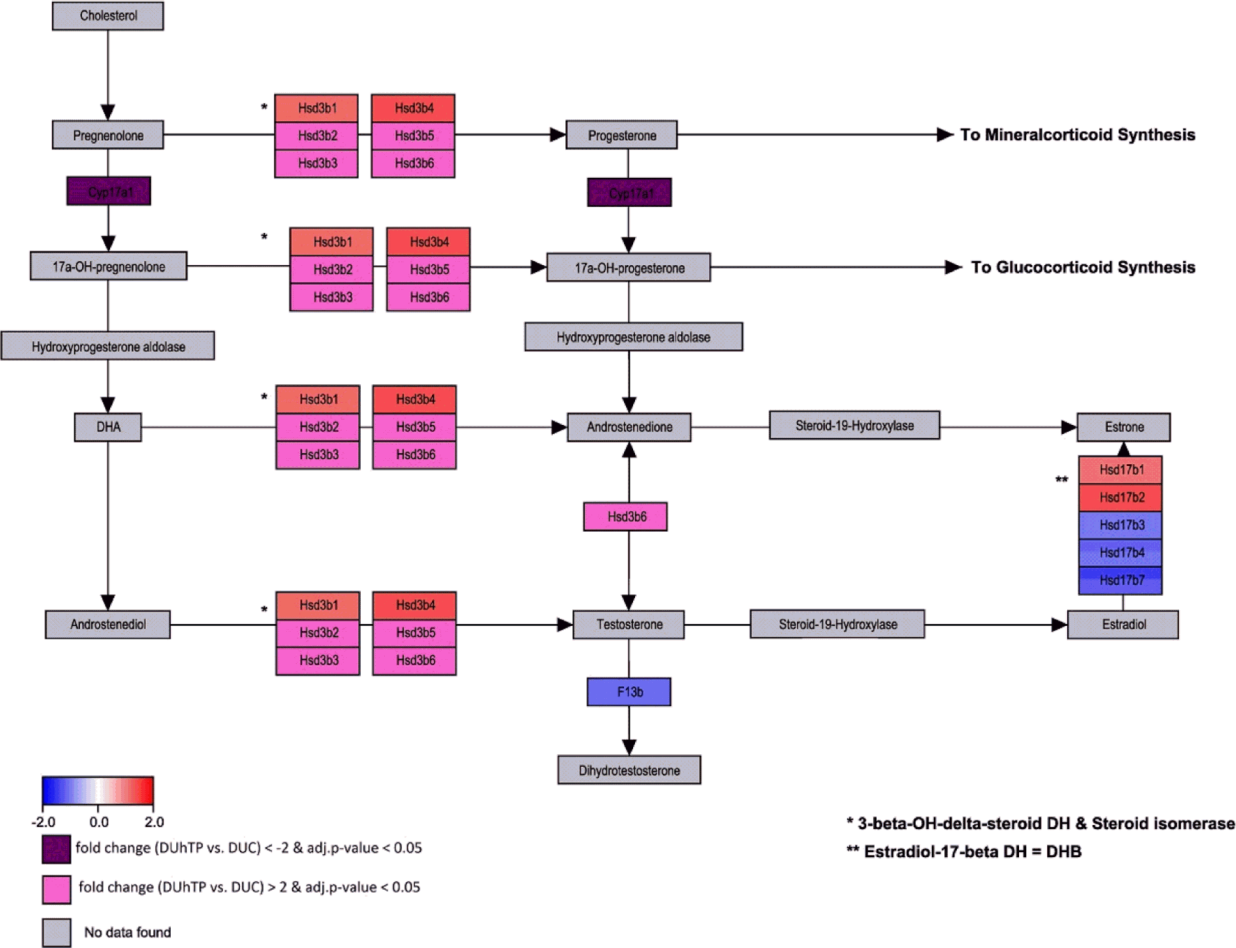
Pathway analysis of microarray results related to Steroid Biosynthesis (Mus musculus, WikiPathway WP55) in the liver of DUhTP versus DUC mice. Enzymatic reactions are marked by arrows. Enzyme encoding genes in boxes are colored red or blue, respectively, to indicate up- or down-regulation in DUhTP compared to control DUC. Different colors in a box for the same gene indicate various probe sets with differing expression. Genes with fold change >2 are colored in pale and dark magenta for up- or down-regulation, respectively.

The gene set of WikiPathway *Steroid Biosynthesis* includes a total of 15 genes whereof 13 were measured. Abundance was significantly different in livers from DUhTP and DUC mice for seven mRNA sequences (Fig 4). The strongest increase of mRNA transcript abundance was found for steroid delta isomerase-5 (Hsd3b5, FC 4.97) and a major decrease was identified for steroid 17-alpha-monooxygenase (Cyp17a1, FC -11.6).

### Quantification of steroids in DUhTP mice versus controls

**Fig 5:**
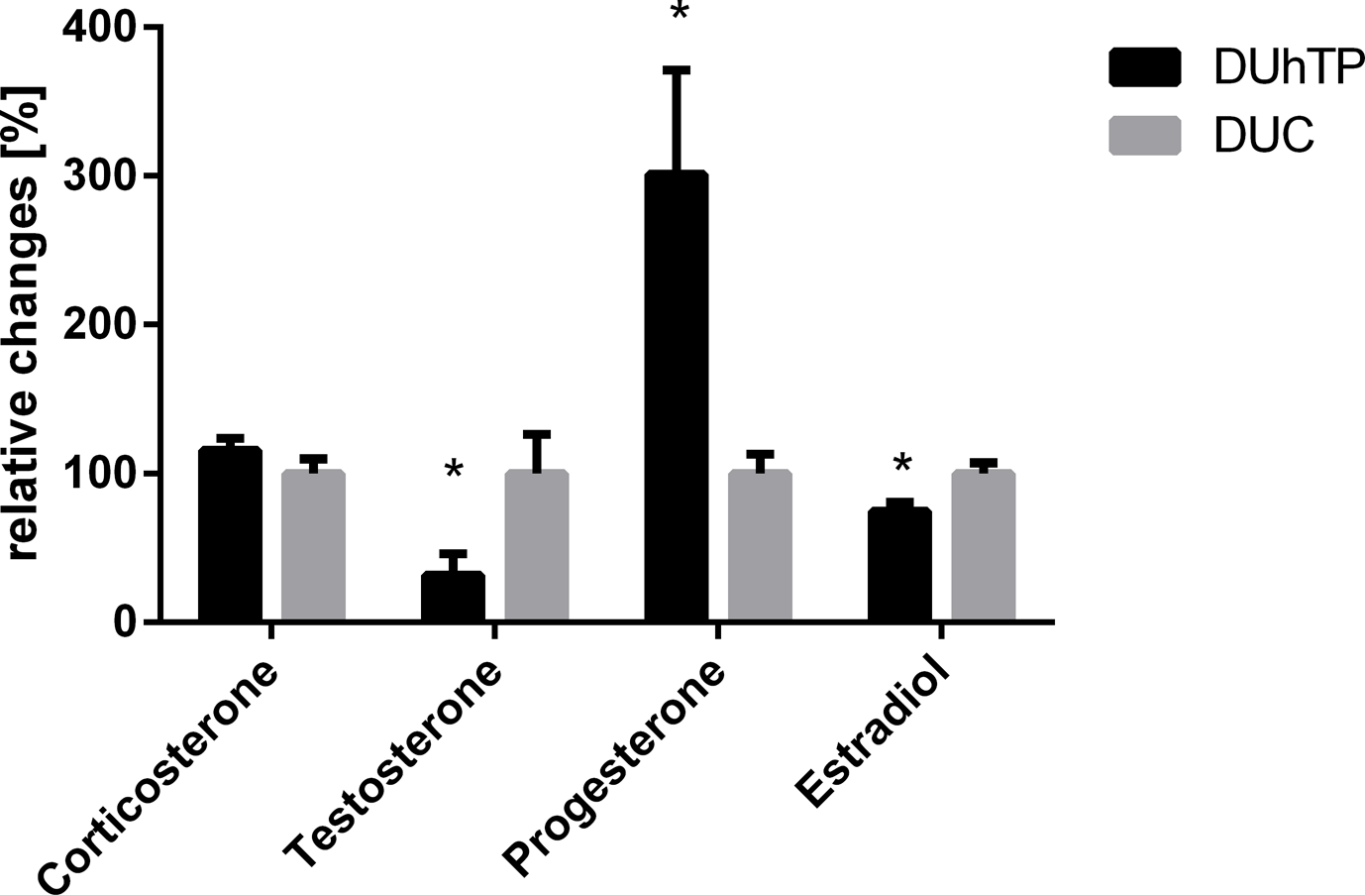
Serum levels of steroids measured in DUhTP and DUC (mean±SEM; N>22; *p<0.05).

Serum levels of progesterone, corticosterone, testosterone and estradiol were analyzed by mass spectrometry in DUhTP and control mice. Progesterone was significantly (p<0.009) increased, while estradiol (p<0.017) was decreased in DUhTP mice compared to controls. Testosterone (p<0.055) was decreased with borderline significance in DUhTP mice. Serum levels of corticosterone were similar in both mouse lines. Serum steroid hormone levels are shown in Fig 5.

## Discussion

In more than 90 rounds of phenotype-selection for high running performance [21] DUhTP mice, also termed marathon mice, are supposed to acquire a complex set of genetically fixed mechanisms enabling superior endurance performance. The marathon mouse model used in the present study is characterized by marked accumulation of body and organ fat as well as by higher concentrations of carbohydrates and lipids in the liver [20]. Here we hypothesized that the metabolomic phenotype of DUhTP mice in the liver may be explained at the level of hepatic mRNA transcript abundance. In order to test this hypothesis, network modeling of hepatic mRNA transcript abundance analyzed in DUhTP mice and unselected controls was performed.

### Induction of glycolysis and inhibition of gluconeogenesis

Metabolomic profiles of liver extracts uncovered large-scale upregulation of carbohydrate metabolism as indicated by strong increases of carbohydrates and in particular different hexose phosphates in DUhTP mice versus controls [20]. Glucose uptake from blood into the liver is mainly achieved by the low affinity glucose transporter GLUT2 (*Slc2a2*), acting independently of insulin, but dependent on the level of blood glucose [38, 39]. In DUhTP mice, abundance of Slc2a2 mRNA is significantly increased compared to unselected controls. Also, mRNA transcript abundance of other low affinity glucose carriers (GLUT3 and GLUT5) tended to be increased in the liver of DUhTP mice. In rats, moderate daily exercise decreased hepatic GLUT expression [40] and this reduction was discussed in the context of a leaner phenotype from daily exercise. Thus, elevated GLUT2 mRNA concentrations in DUhTP mice are correlated with the obese phenotype. Abundance of mRNA coding for the ubiquitous insulin-dependent glucose transporter Slc2a1 (GLUT1), with a high affinity for glucose ensuring permanent glucose uptake, is decreased in DUhTP mice. Exercise-related modulation of GLUT1 expression in the liver has not yet been reported in the literature.

As an initial step of glycolysis, glucokinase (also hexokinase 4, GCK) is responsible for conversion of glucose to glucose-6-phosphate [41, 42]. Abundance of liver-specific GCK mRNA is almost 3-fold increased in DUhTP mice (p<0.005). GCK expression is upregulated by insulin [43, 44] as a mechanism of glucose homeostasis. However, in DUhTP mice, insulin concentrations in blood are unaltered by the genotype [20] and thus do not explain higher abundance of GCK mRNA in the present model. Notably, physical activity reduced hepatic activity of GCK in rats [45]. Higher levels of GCK mRNA expression and increased protein expression of GCK have been discussed in a context of hepatic energy metabolism before [20]. Abundance of transcription factor SREBP1 mRNA, is strongly increased in DUhTP compared to control mice (FC 2.81, p<0.005). Acting as a transcription factor for glucokinase [46], SREBP1 may increase expression of GCK mRNA also in DUhTP mice. Ruiz and colleagues [47] have shown that RNAi knockdown of hepatic *Srebp1* in mice increased phosphoenolpyruvate-carboxykinase (PEPCK, Pck1) expression, in accordance with elevated mRNA levels of SREBP1 and decreased levels of Pck1 mRNA in DUhTP mice. On the level of mRNA expression, it is concluded that glycolysis is increased, whereas gluconeogenesis is decreased in the liver of DUhTP mice at an age of 10 weeks. Because previous findings in 7-week DUhTP mice revealed higher PEPCK protein expression in the liver from DUhTP mice suggesting increased gluconeogenesis [20], there is, however, the possibility of age-specific effects on metabolic regulation in the liver of DUhTP mice.

### Triacylglyceride synthesis in DUhTP mice

Abundance of ATP-citrate lyase mRNA (ACLY) and fatty acid synthetase mRNA (FAS; FC 1.75, p<0.05) were significantly increased in DUhTP (FC 1.69) versus control mice. Hepatic expression of FAS is suppressed by physical activity [48]. Consequently, increased abundance of FAS mRNA in the liver may be a specific adaption of metabolic control in DUhTP mice. Both FAS [49] and ACLY [50] are regulated by the transcription factor SREBP1. Because SREBP1 was also discussed in the context of carbohydrate metabolism (GCK and Pck1), it may act on different metabolic pathways in the liver of DUhTP mice. In the liver, SREBP1 is a potent activator of fatty acid synthesis [51]. As demonstrated previously [20], DUhTP mice are characterized by more than a 2-fold increase in triglyceride content in the liver. SREBP1 is not known to upregulate enoyl-CoA-hydratase domain-containing 3 (Echdc3, FC 1.71) and peroxisomal trans-2-enoyl-CoA reductase (Pecr, FC 1.71), two additional lipogenic enzymes that are more abundant in DUhTP versus control mice on the levels of mRNA. Hence we may speculate that higher expression levels of different lipogenic enzymes represent polygenic adaptations established during long-term selection of DUhTP mice. Altogether, results from microarray analysis suggest increased biosynthesis of triglycerides in the liver of DUhTP mice, which is in line with results from a metabolome study, performed in the liver of DUhTP mice before [20].

### Cholesterol and steroid biosynthesis

Results from mRNA analysis further support results from the previous metabolome study on hepatic extracts from DUhTP mice [20] and unpublished data that demonstrated higher levels of stigmasterol and cholesterol in DUhTP mice versus controls. Notably, a number of mRNA transcripts that are present at higher concentration in DUhTP versus control mice (e.g. Fdps, Idi1, Sqle, Dhcr7) are transcriptionally activated by SREBP-1 or SREBP-2 [51]. Thus, in addition to carbohydrate and fatty acid metabolism, as discussed before, SREBPs could act as important regulatory factors also for cholesterol synthesis in the liver of DUhTP mice [51]. Follow-up studies will have to address the specific function of different SREBP-family members in the liver of DUhTP mice.

Analysis of mRNA expression with respect to steroid hormone production in the liver of DUhTP mice revealed lower production of glucocorticoids and sex steroids, but increased production of mineralocorticoids. Interestingly, direct steroid hormone measurement in serum of DUhTP and control mice supports results from the mRNA expression analysis related to the steroid hormone biosynthesis pathway. By the inhibition of steroid 17-alpha-monooxygenase (CYP17a1) mRNA, expression of the 17-hydroxy forms of pregnenolone and progesterone could be repressed in DUhTP mice, potentially resulting in higher progesterone and lower sex steroid concentrations present in DUhTP mice versus controls. In mammalian testes, CYP17a1 is increased by exercise [52]. However, the relevance of hepatic steroid production is unclear, although steroidogenic enzyme expression by the fetal liver has been discussed as an effector of circulating steroid levels [53]. Nevertheless, steroids are considered as potent effectors of physical exercise performance and it has been demonstrated that progesterone, testosterone or estradiol are regulated also in response to acute physical exercise in human subjects [54–56].

### Conclusions

Over-representation analysis with PathVisio of biochemical pathways suggested increased activity of the glycolytic pathway as well as increased production of fatty acids and cholesterol in the liver of DUhTP mice and therefore confirmed previous results on the level of metabolome or proteome. A high number of mRNA transcripts present at increased abundance in the liver from DUhTP mice versus controls are regulated by SREBPs. We may thus assume important functions of SREBPs for metabolic adaptations present in the liver of DUhTP mice. Analysis of hepatic mRNA expression further indicated higher progesterone synthesis and reduced production of sex steroids in the liver of DUhTP mice versus controls. In serum samples from DUhTP mice and controls, both predictions were supported on the metabolic level by current mass spectrometry.

## Supporting Information

S1 Table Primers used in the present study. (DOC)

S2 Table Results of qRT-verification of liver mRNA expression in DUhTP and DUC. Listed are transcript numbers with standard error (SE) plus fold change (FC) between both strains and associated p-value of an unpaired t-test.
(DOC)
