# Supplementary material for: Advanced Running Performance by Genetic Predisposition in Male Dummerstorf Marathon Mice (DUhTP) Reveals Higher Sterol Regulatory Element-Binding Protein (SREBP) Related mRNA Expression in the Liver and Higher Serum Levels of Progesterone
Source: PLoS One. 2016 Jan 22;11(1):e0146748. doi: 10.1371/journal.pone.0146748 (PMC4723140; doi:10.1371/journal.pone.0146748)
Supplement: S1 Table — (DOC) [file pone.0146748.s001.doc]

**S1 Table** Primers used in the present study

| Gene title | Gene-ID | Symbol | Primer pair sequence 5´- 3´ |
| --- | --- | --- | --- |
| aldo-keto reductase family 1 | 11677 | Akr1b3 | ccattcagaggaacttggtag  tggaaggggtaatccttgtg |
| calcium/calmodulin-dependent protein kinase II, beta |  | Camk2b | AATCTTCCGACAGCACCAAC  CTGTGGAAATCCATCCCTTC |
| cytochrome c oxidase subunit VIb polypeptide 2 | 333182 | Cox6b2 | gcaggaattgctacttggatg  agtactcgcagggttgtgtg |
| cytochrome P450 2B9 | 13094 | CYP2b9 | ggaaaccaggccattggtag  tggactgttgggaggaagag |
| cytochrome P450 2D13 | 68444 | CYP2D13 | tccaattctcctgcacatcc  ccctttgccttctccatttc |
| cytochrome P450, family 51 |  | Cyp51 | TCCTGGCAGAGTTGTGACTT  CAGCCTCTGCTGTGTTGTTC |
| glutathione S-transferase, mu 3 | 14864 | Gstm3 | cacagcccttttctgcaatc  ggcactcgagtattgacctt |
| heat shock protein 110 | 15505 | HSP110 | gtcagggtcctgtggagttg  acacacatgcggaaatgaag |
| heat shock protein 1A | 193740 | Hspa1a | atgcttgtgtcgggtcctt  acccgagttcaggatggtt |
| histocompartibility 2, class II antigen E alpha | 14960 | H2-Ea | tgacaagttctcccctcca  aaccccagtgatccacctc |
| histocompatibility 2, K1, K region | 14972 | H2-K1 | CTGCCTGGAGTGGACTTGG  GCTGTGGAAGGGAAGACAGA |
| iodothyronine Dioinase type1 | 13370 | Dio1 | gcattggaaacacttttctgg  gcctgcgatttggtttagtt |
| solute carrier family 26 a1 | 231583 | Slc26a1 | tccaccacctcagcatctc  ggctgaccagtacgacctt |
| squalene epoxidase | 20775 | Sqle | gctgaacaaatttacccaca  caactgtcattcctccacca |
| zinc finger protein 236 | 329002 | Zfp236 | ttatacgcctgggatgtgg  ttccttggcttttggtttg |
