# Supplementary material for: Advanced Running Performance by Genetic Predisposition in Male Dummerstorf Marathon Mice (DUhTP) Reveals Higher Sterol Regulatory Element-Binding Protein (SREBP) Related mRNA Expression in the Liver and Higher Serum Levels of Progesterone
Source: PLoS One. 2016 Jan 22;11(1):e0146748. doi: 10.1371/journal.pone.0146748 (PMC4723140; doi:10.1371/journal.pone.0146748)
Supplement: S2 Table — Listed are transcript numbers with standard error (SE) plus fold change (FC) between both strains and associated p-value of an unpaired t-test. (DOC) [file pone.0146748.s002.doc]

S2Table  **Results of qRT-verification of liver mRNA expression in DUhTP and DUC.** Listed are transcript numbers with standard error (SE) plus fold change between both strains and associated p-value of unpaired t-test.

| Gene Symbol | Transcript number (±SE)  DUhTP | Transcript number (±SE)  DUC | Fold change | p-value |
| --- | --- | --- | --- | --- |
|  |  |  |  |  |
| Akr1b3 | 4.60E+02 (±1.01E+02) | 2.04E+02 (±5.39E+01) | 2.18 | 0.0322 |
| Camk2b | 3.63E+02 (±5.40E+01) | 2.18E+02 (±3.40E+01) | 1.50 | 0.0450 |
| Cox6b2 | 5.28E+02 (±1.78E+02) | 2.19E+01 (±5.56E+00) | 18.59 | 0.0082 |
| Cyp2b9 | 9.58E+02 (±2.01E+02) | 5.98E+00 (±1.84E+00) | 177.54 | 0.0002 |
| Cyp2d13 | 6.92E+02 (±1.07E+02) | 1.05E+03 (±2.99E+02) | -1.52 | 0.2856 |
| Cyp51 | 4.59E+01 (±7.72E+00) | 3.10E+01 (±1.44E+01) | 2.73 | 0.0067 |
| Dio1 | 5.18E+02 (±9.90E+01) | 1.37E+02 (±2.10E+01) | 3.79 | 0.0016 |
| Gstm3 | 1.33E+03 (±2.31E+02) | 2.83E+02 (±7.59E+01) | 6.78 | 0.0005 |
| Hsp110 | 1.61E+02 (±2.75E+01) | 3.36E+02 (±1.05E+02) | -1.51 | 0.1424 |
| Hspa1a | 1.21E+02 (±5.71E+01) | 8.26E+02 (±3.62E+02) | -6.81 | 0.0753 |
| H2-Ea | 1.41E+03 (±1.81E+02) | 1.70E+02 (±5.56E+01) | 8.28 | 0.0000 |
| H2-K1 | 7.01E+03 (±1.03E+03) | 7.72E+03 (±9.68E+02) | 1.02 | 0.7550 |
| Slc26a1 | 1.28E+03 (±1.80E+02) | 9.36E+02 (±3.71E+02) | 2.14 | 0.0097 |
| Sqle | 1.45E+02 (±3.09E+01) | 4.98E+01 (±1.80E+01) | 2.75 | 0.0132 |
| Zfp236 | 2.17E+02 (±3.94E+01) | 1.66E+02 (±2.54E+01) | 1.19 | 0.2759 |
